# Simple and Economical Extraction of Viral RNA and Storage at Ambient Temperature

**DOI:** 10.1128/spectrum.00859-22

**Published:** 2022-06-01

**Authors:** Sarah Hernandez, Fátima Cardozo, David R. Myers, Alejandra Rojas, Jesse J. Waggoner

**Affiliations:** a Emory University, Department of Medicine, Division of Infectious Diseases, Atlanta, Georgia, USA; b Universidad Nacional de Asunción, Instituto de Investigaciones en Ciencias de la Salud, Departamento de Salud Pública, San Lorenzo, Paraguay; c Wallace H. Coulter Department of Biomedical Engineering, Emory University and Georgia Institute of Technology, Atlanta, Georgia, USA; d Aflac Cancer and Blood Disorders Center of Children’s Healthcare of Atlanta, Department of Pediatrics, Emory University, Atlanta, Georgia, USA; e Universidad Nacional de Asunción, Instituto de Investigaciones en Ciencias de la Salud, Departamento de Producción, San Lorenzo, Paraguay; f Rollins School of Public Health, Department of Global Health, Atlanta, Georgia, USA; Center for Research and Advanced Studies (CINVESTAV-IPN)

**Keywords:** ribonucleic acid, extraction, dengue virus, molecular testing

## Abstract

RNA extraction is essential for the molecular detection of common viral pathogens. However, available extraction methods and the need for ultra-cold storage limit molecular testing in resource-constrained settings. Herein, we describe the development of an economical RNA Extraction and Storage (RNAES) protocol that eliminates requirements for instrumentation, expensive materials, and preserved cold chain. Through an iterative process, we optimized viral lysis and RNA binding to and elution from glass fiber membranes included in simple RNAES packets. Efficient viral lysis was achieved with a nontoxic buffer containing sucrose, KCl, proteinase K, and carrier RNA. Viral RNA binding to glass fiber membranes was concentration dependent across seven orders of magnitude (4.0–10.0 log_10_ copies/μL) and significantly increased with an acidic arginine binding buffer. For the clinical evaluation, 36 dengue virus (DENV)-positive serum samples were extracted in duplicate with the optimized RNAES protocol and once in an EMAG instrument (bioMérieux). DENV RNA was successfully extracted from 71/72 replicates (98.6%) in the RNAES protocol, and real-time RT-PCR cycle threshold (*C_T_*) values correlated between extraction methods. DENV RNA, extracted from clinical samples, was stable when stored on dried RNAES membranes at ambient temperature for up to 35 days, with median eluate RNA concentration decreasing by 0.18 and 0.29 log_10_ copies/μL between day 0 and days 7 and 35, respectively. At a cost of $0.08/sample, RNAES packets address key limitations to available protocols and may increase capacity for molecular detection of RNA viruses.

**IMPORTANCE** RNA extraction methods and ultra-cold storage requirements limit molecular testing for common viruses. We developed a simple, flexible, and economical method that simultaneously addresses these limitations. At $0.08/sample, the new RNA Extraction and Storage (RNAES) protocol successfully extracted viral RNA from acute-phase sera and provided stable, ambient-temperature RNA storage for 35 days. Using this approach, we expect to improve RNA virus detection and outbreak response in resource-constrained settings.

## INTRODUCTION

RNA viruses are the largest group of human viral pathogens and the most common cause of emerging infectious disease outbreaks (1, 2). Clinical management and disease containment rely on accurate laboratory diagnosis, and for many RNA viruses, molecular methods provide the most sensitive and specific acute-phase diagnostics (3–7). RNA extraction remains a crucial step in sample preparation that ensures optimal performance of such methods, but extraction presents many challenges owing to the relative instability of RNA compared to DNA and the presence of RNA degrading enzymes (RNases) in clinical samples (8–10). Extraction is generally performed using commercial kits that are costly, rely on proprietary materials, and can be difficult to reliably obtain in resource-constrained settings or emerging markets (11, 12). Kits often require the use of dedicated instruments, corrosive and hazardous chemicals, and −80°C storage of the resulting eluate if testing will not be performed within 24 hours (8–10, 13, 14). As a result, RNA extraction and storage remain major barriers to the implementation and use of molecular methods.

Arboviruses comprise the subset of RNA viruses transmitted by infected arthropod vectors such as mosquitoes and ticks. These have resulted in large, recent outbreaks caused by the introduction of viruses into naïve populations (e.g., chikungunya virus [CHIKV] and Zika virus [ZIKV] in the Americas in 2014–2016) (15–18) or reemergence of viruses in populations residing in endemic regions (e.g., yellow fever virus [YFV] and dengue virus [DENV]) (19–23). Of the arboviruses, DENV is responsible for the greatest burden of human disease, causing an estimated 100 million symptomatic infections (dengue cases) per year spread over 125 countries (24). Dengue presents with nonspecific, systemic symptoms that cannot be clinically differentiated from other causes of an acute febrile illness, and diagnostic confirmation relies on laboratory test availability (7, 20). However, in endemic countries such as Paraguay, DENV causes large seasonal outbreaks that exhaust laboratory reagent supply and testing capacity, resulting in under detection and potentially worse clinical outcomes (4, 25, 26).

In this study, we developed a simple RNA Extraction and Storage (RNAES) protocol for use with serum and plasma in resource-constrained settings. This economical method utilizes a biosafe viral lysis buffer and capillary flow across an RNA binding membrane in simple packets to yield RT-PCR-compatible RNA and provide stable RNA storage at ambient temperatures. The protocol was developed and optimized using contrived DENV-positive clinical samples and purified arboviral RNA. Clinical evaluation was then performed on a set of 36 acute-phase samples from confirmed dengue cases in Paraguay.

## RESULTS

### Viral lysis.

Of the four experimental lysis buffers evaluated (deionized water, STET, sodium dodecyl sulfate (SDS)-NaCl, and sucrose buffer), SDS-NaCl and sucrose solutions performed similarly, yielding earlier DENV cycle threshold (*C_T_*) values (indicating increased RNA yield) by real-time reverse transcriptase PCR (rRT-PCR) (Table S1 in supplemental materials). To eliminate potential SDS inhibition of downstream molecular testing, sucrose buffer was chosen as the lysis buffer for further experiments. To further enhance RNA recovery and prevent degradation, varying amounts of poly-A carrier RNA and proteinase K were added to the lysis buffer. In side-by-side comparisons, carrier RNA (2.5 μg/sample) and proteinase K (5.0 μg/sample) significantly increased RNA recovery (Tables S2 and S3, respectively). Higher concentrations of carrier RNA (5 μg/sample; Table S2) and proteinase K (10 μg/sample; Table S3) in the lysis mixture did not enhance RNA recovery. In addition, the impact of lysis incubation on RNA recovery at room temperature was tested at various time durations from 10 to 60 minutes. Samples were stable for up to 1 hour in lysis mixture, but longer incubation times did not result in increased RNA recovery after 10-minute incubation at room temperature (Table S4).

### Membranes.

**FIG 1:**
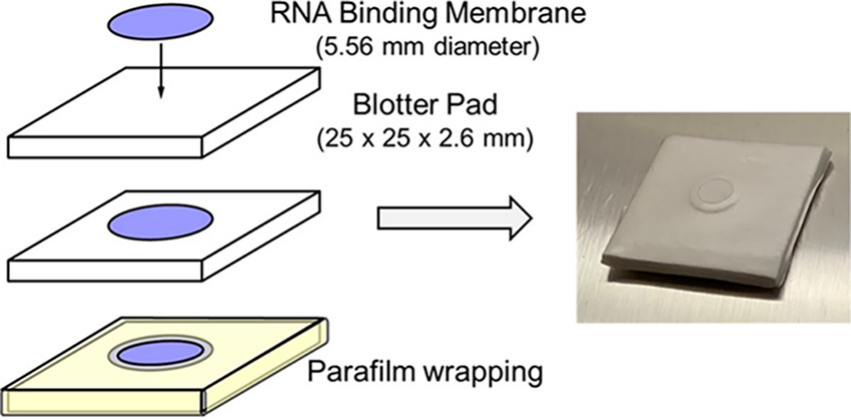
Components and assembly of RNA Extraction and Storage (RNAES) packets (left) and an assembled packet (right). Whatman 3, Fusion 5, and GF/D were evaluated as RNA binding membranes.

Extraction packets were assembled as shown in Fig. 1 using Whatman 3, Fusion 5, and GF/D RNA binding membranes. To compare RNA recovery from the different membranes, 15 μL of purified DENV, CHIKV, and OROV RNA were mixed with lysis buffer and ethanol and added to the packets. RNA was then eluted and tested by rRT-PCR. RNA recovery was concentration dependent (Table 1; Fig. S1) and successful for all viruses and concentrations on the GF/D membranes (12/12). One extraction failed with both the Whatman 3 and Fusion 5 membranes (11/12 each; Table 1). *C_T_* values were lowest for RNA recovered from GF/D membranes, and based on these data, the GF/D membrane was selected for inclusion in the final RNAES packet.

**TABLE 1:** rRT-PCR *C_T_* values for RNA of different arboviruses following binding to and elution from Whatman 3, Fusion 5, and GF/D membranes^*a*^

Viral RNA	Whatman 3	Fusion 5	GF/D
DENV sample 1	30.13	30.51	30.13
	31.49	30.32	30.64
DENV sample 2	38.20	37.18	36.84
	—^*b*^	38.52	36.44
CHIKV sample 1	27.07	27.71	25.05
	25.61	28.38	24.89
CHIKV sample 2	35.34	—^*b*^	34.02
	35.98	40.80	38.04
OROV sample 1	22.34	23.86	22.97
	23.21	23.22	22.87
OROV sample 2	31.22	33.32	32.75
	31.12	33.01	31.50

a rRT-PCR, real-time reverse transcriptase PCR; *C_T_*, cycle threshold; CHIKV, chikungunya virus; DENV, dengue virus; OROV, Oropouche virus.

b Extraction failed, no *C_T_* value.

### Amino acid binding buffers.

**FIG 2:**
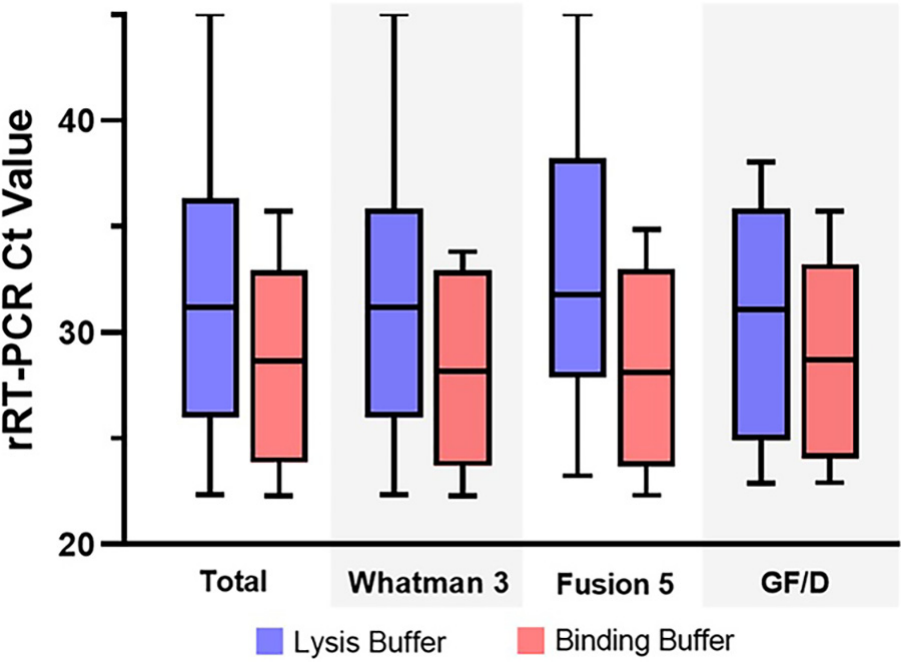
Amino acid binding buffers improve RNA yield from RNAES packets. Arginine binding buffer resulted in lower and more consistent real-time reverse transcriptase PCR (rRT-PCR) cycle threshold (*C_T_*) values (improved RNA yield) following chikungunya virus (CHIKV), dengue virus (DENV), and Oropouche virus (OROV) RNA binding to and elution from extraction packets assembled with Whatman 3, Fusion 5, and GF/D membranes. Box-and-whisker plots display median and range of all values.

Arginine- and glutamine-based amino acid binding buffers were assessed as a method to modulate RNA-membrane interactions when the lysate was loaded on the packet. Contrived DENV-positive serum samples were lysed and then treated with either amino acid buffer. Arginine treatment yielded lower *C_T_* values overall and decreased variability compared to glutamine, although results did not reach statistical significance (Fig. S2). To further evaluate the impact of arginine binding buffer, DENV, CHIKV, and OROV RNA were added to RNAES packets in either sucrose lysis buffer or arginine buffer (plus ethanol in both cases). Arginine buffer demonstrated lower mean *C_T_* values and decreased variability in *C_T_* values with each membrane (Fig. 2).

**FIG 3:**
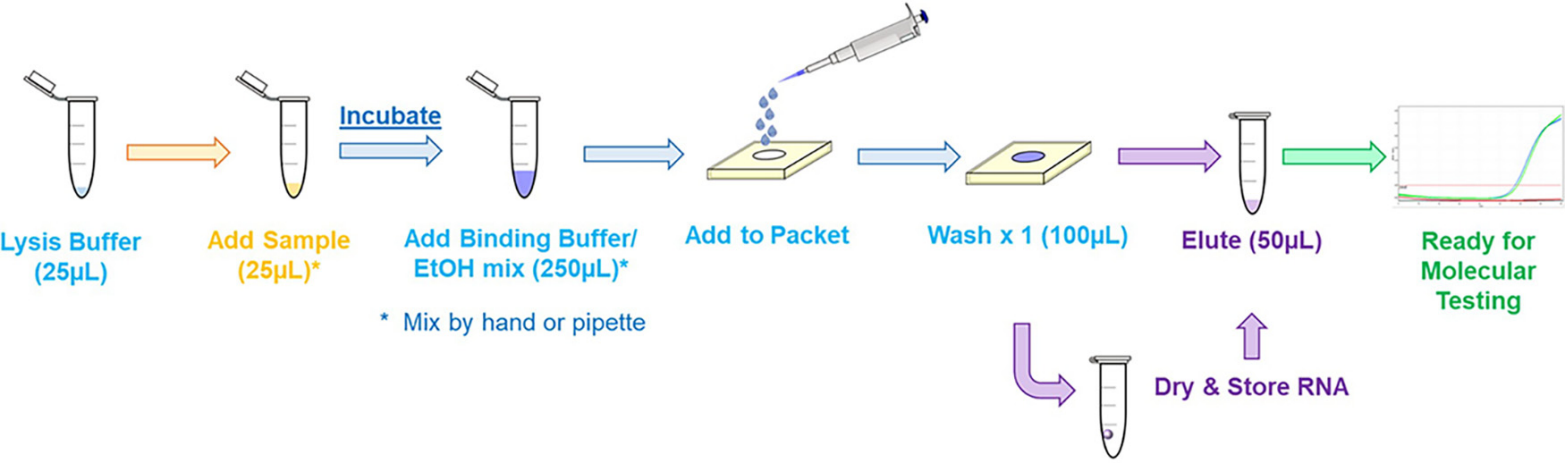
Optimized RNAES workflow. For elution, membranes are incubated in TE buffer for 1 minute and then removed and discarded. All steps are performed at ambient temperature.

Evaluation of arginine buffer as a viral lysis buffer with contrived DENV samples resulted in worse RNA recovery compared to sucrose buffer (Fig. S3A). The arginine buffer was subsequently integrated into the final RNAES protocol as a binding buffer mixed with ethanol (Fig. 3). This allowed for both successful viral lysis and improved binding of viral RNA to the packet membrane (Fig. S3B). With the addition of the arginine buffer to the procedural workflow, MgCl_2_ in the sucrose buffer was changed to KCl to harmonize buffer preparations, which had no impact on RNA recovery (Table S5).

### Clinical evaluation.

**FIG 4:**
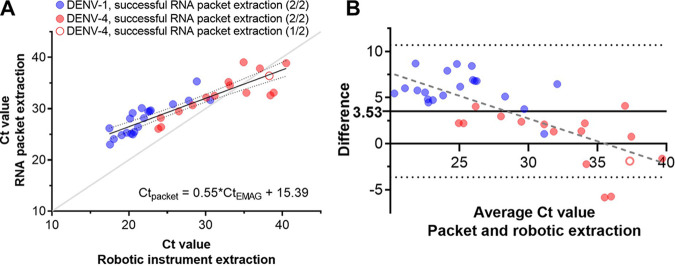
DENV RNA was successfully extracted from clinical samples using the economical RNAES protocol, and rRT-PCR *C_T_* values correlated with results following EMAG robotic extractions. Successful RNA extraction was defined as a positive result in the DENV multiplex rRT-PCR (42, 43). (A) Average *C_T_* values from duplicate packet extractions are graphed versus the *C_T_* following EMAG extraction for 36 serum samples positive for DENV-1 (*n* = 20) and DENV-4 (*n* = 16). Seventy-one out of seventy-two eluates (98.6%) from the packets had detectable DENV RNA. The sample from which 1 of 2 replicates was positive is displayed as a half-filled circle. Results of the linear regression of packet vs. EMAG *C_T_* are shown along with the line of identity (light gray line). Dotted lines show 95% confidence interval of the best-fit line. (B) Bland-Altman plot of the comparison between packet and EMAG *C_T_* values. Dashed line represents the linear regression of the differences.

Demographic information and laboratory data for the 36 DENV-positive serum samples selected for the clinical evaluation are shown in Table 2. Serum viral loads ranged from 4.73 to 8.22 log_10_ copies/mL. When extracted in duplicate with the RNAES protocol (72 total extractions), DENV RNA was detected in 71/72 replicates (98.6%), and DENV multiplex rRT-PCR *C_T_* values correlated with results following EMAG extraction (Fig. 4A; Table S6). When compared to a commercial extraction system, extraction with RNAES protocol demonstrated improved efficiency as sample concentration decreased (Fig. 4B).

**TABLE 2:** Clinical and DENV laboratory data for 36 clinical samples extracted with the RNAES protocol^*a*^

Category	Result
Total, *n*	36
Gender, female, *n* (%)	21 (58.3)
Age, mean (SD)	28.9 (14.1)
Day postsymptom onset, mean (SD)	3.6 (1.6)
Serotype, *n* (%)	
DENV-1	20 (55.6)
DENV-4	16 (44.4)
Viral load, mean (SD)^*b*^	7.1 (1.4)

a RNAES, RNA Extraction and Storage.

b Viral load expressed as log_10_ copies/mL serum.

**FIG 5:**
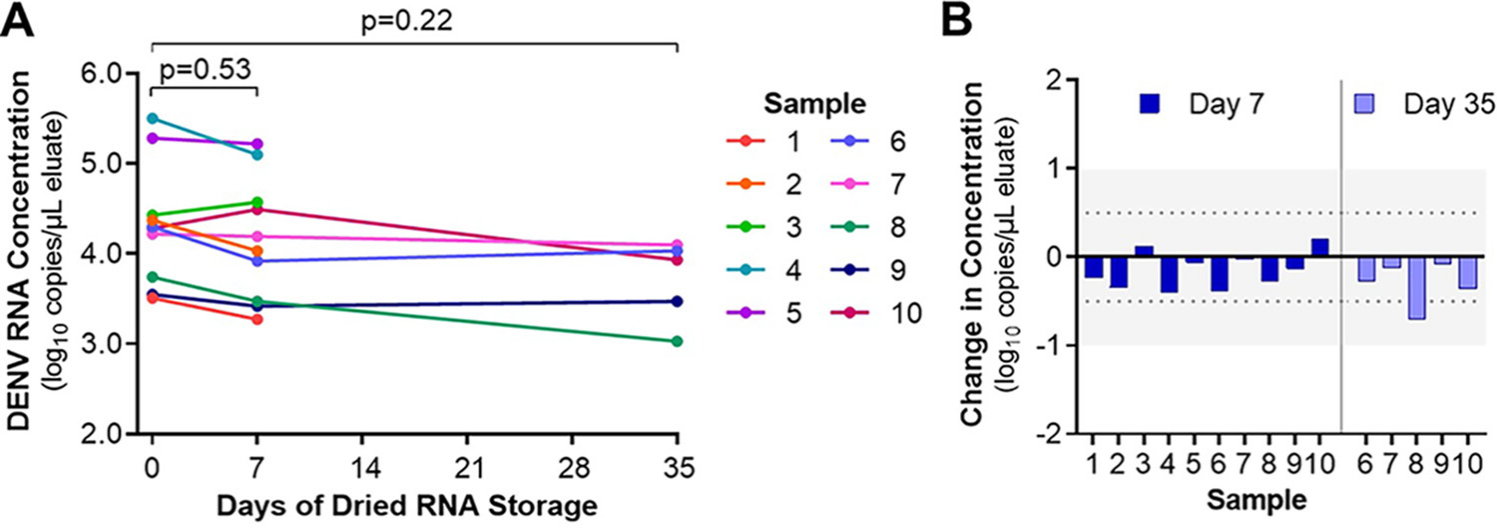
DENV RNA stored on dried extraction membranes is stable at ambient temperature for up to 35 days. (A) Average DENV RNA concentration in eluates from RNAES packets performed in duplicate is displayed for 10 clinical samples that were fully extracted on day 0 or that underwent lysis, addition to GF/D membrane-containing packets, glycine wash, drying, and storage at ambient temperature. RNA was eluted off the dried membranes and tested by rRT-PCR on days 7 and 35. DENV RNA concentration was calculated from a 4-point standard curve included on each run. (B) Change in RNA concentration between day 0 and days 7 and 35. The shaded region highlights the expected intra-run variability of rRT-PCR (±0.5 log_10_ copies/μL).

A subset of DENV-positive samples was then selected for evaluation of RNA stability when stored on GF/D membranes at room temperature for 7 days (*n* = 10 samples) and 35 days (*n* = 5 samples). RNA was eluted on day 0 or dried on the RNA binding membrane after the glycine wash and eluted on day 7 or day 35. DENV RNA concentration in the eluates was calculated in the DENV multiplex rRT-PCR. Concentrations in the eluates did not differ significantly across time points (Fig. 5A; Table S7). Median DENV RNA concentration in the eluates was 4.29 log_10_ copies/μL on day 0 and 4.11 log_10_ copies/μL on day 7. For the day 35 analysis, median concentrations were 4.22 log_10_ copies/μL on day 0 and 3.93 log_10_ copies/μL on day 35. DENV RNA concentration fluctuated between time points but remained within the expected error for quantitative rRT-PCR for all but one sample (±0.5 log_10_ copies; Fig. 5B).

## DISCUSSION

This study presents the development of the RNAES protocol. This is an alternative, low-cost extraction method that yields RNA compatible with downstream rRT-PCR analysis and is suitable for implementation in resource-constrained settings as it eliminates the need for expensive proprietary materials, hazardous chemicals, or electricity during the extraction process. The final protocol has an estimated cost of USD $0.08 per sample and successfully detected 98.7% of DENV-positive clinical samples when compared to a commercial robotic extraction system (EMAG) with an estimated cost of USD $8.00 per sample, plus a one-time instrument cost of USD $115,000. Clinical samples were collected during two large DENV outbreaks in Paraguay (DENV-1 in 2018; DENV-4 in 2019–2020). Notably, the range of viral loads in samples that were available for extraction in the current study represents 92.7% of all DENV-positive samples with quantifiable viral loads from these two large outbreaks (Ref. 4, DENV-1; A. Rojas and J.J. Waggoner unpublished observations, DENV-4). Samples with viral loads outside of this range were no longer available for extraction.

The RNAES protocol maintained sensitivity as viral load decreased, with a smaller difference in *C_T_* values compared to the EMAG as the *C_T_* value increased. This cause for the increase in extraction efficiency at lower concentrations in the RNAES protocol is not clear but could result from saturation of the membrane at higher viral loads due to binding of viral RNA in addition to other RNAs and proteins in complicated clinical samples. These data demonstrate the preserved performance of the RNase protocol at low viral loads and indicate the potential utility of this protocol in a real-world testing scenario outside of a high resource research laboratory.

In addition to examining the limitations posed by RNA extraction methods, in this study we address the temperature constraints of common RNA storage procedures. Ultra-cold storage of samples and eluates is costly to maintain, has limited capacity, and complicates the shipment of samples from collection sites to reference laboratories (8, 13, 14). Successful ambient-temperature RNA storage has previously required lyophilization, encapsulation in steel or silica, or both (8, 27). However, these are not realistic options for widespread implementation. We report an alternative technique where viral RNA is simply air dried on the extraction membrane and integrity is maintained for rRT-PCR analysis. Success likely results from washing with highly acidic buffers and membrane drying to reduce RNA transesterification and RNase activity (28, 29). This technique resulted in RNA stability for up to 35 days. The RNAES protocol, therefore, provides a robust option for both RNA extraction and ambient-temperature storage.

Various efforts to provide a sustainable alternative to expensive commercial RNA extraction kits utilize magnetic bead technology or solid-phase extraction methods that include biohazardous reagents (9, 10, 30–34). Magnetic bead technologies provide adjustable surface chemistries and ease of use for nucleic acid isolation (33). However, beads are relatively expensive and may be difficult to acquire and implement in resource-constrained settings. Other solid phase alternatives rely on toxic phenol and/or guanidine-based solutions that inhibit downstream molecular testing and require special handling in the laboratory (30, 31, 35). To both reduce inhibitory reagents in the procedure and utilize safer reagents for distribution outside of standard molecular laboratories, we assessed an alternative sucrose-based lysis buffer, originally inspired by work in botany (36). Sucrose solutions have been used in various DNA and RNA isolation protocols to increase nucleic acid yield and RNA stability without compromising testing integrity (36, 37). Combined with low-cost extraction packets, the sucrose lysis buffer yields a simple alternative RNA extraction protocol that utilizes accessible and safe reagents suitable for the field setting.

Amino acid buffers were also examined as alternative lysis solutions, as a previous study found that several amino acid solutions improved nucleic acid recovery by modulation interactions between RNA and silica membranes (38). Positively charged arginine and polar uncharged glutamine were chosen for evaluation with RNAES packets due to their distinct chemical properties. Although both amino acid buffers improved RNA yield, this did not translate to improved DENV RNA recovery from contrived samples when arginine buffer was used as a lysis solution. A combined protocol with an arginine binding buffer after lysis in sucrose buffer leveraged the properties of both solutions. These data demonstrate the applicability of amino acid buffers in RNA extraction protocols and highlight the importance of rigorous clinical evaluation for analytical findings obtained under optimal laboratory conditions.

Limitations of the current study include the use of serum and plasma with the extraction packets. These typically require centrifugation for preparation, although represent the most common specimen types for DENV diagnostic testing (4, 7, 13, 39, 40). The use of carrier RNA and proteinase K within the lysis mixture also require cold storage following reconstitution, but both reagents can be shipped and stored in a lyophilized format. To address these limitations, future studies should evaluate adaptations to the extraction packets for use with whole blood and alternative methods for ambient temperature storage of carrier RNA and proteinase K.

In conclusion, we combined methods inspired by nucleic acid isolation protocols used in botany and analytical chemistry with capillary-driven RNA separation to create the low-cost, flexible, and widely accessible RNAES protocol. This represents a unique solution to the demands of extracting and storing viral RNA with the potential to expand capacity for molecular testing and increase pathogen detection.

## MATERIALS AND METHODS

### Packet design.

Basic mechanical design of the packet was based on a method for DNA extraction (41). Packets were prepared with a 5.56-mm diameter membrane disk sandwiched between a square blotter pad base (25 × 25 × 2.6 mm; VWR International, Radnor, PA) and a Parafilm cover with a 3.96-mm diameter opening (Research Products International, Mt. Prospect, IL) centered over the membrane (Fig. 1). Packets were assembled with Whatman 3, Fusion 5, and glass microfiber (GF/D) membranes (all from MiliporeSigma, Burlington, MA).

### Lysis and binding buffers.

Four experimental lysis buffers were evaluated: deionized water, STET (8% sucrose, 5% TritonX-100, 50 mM Tris-HCl, and 50 mM EDTA; Teknova, Hollister, CA), SDS-NaCl (4% SDS, pH 7.5; and 0.5 M NaCl) (30), and a sucrose buffer (50 mM Tris-HCl, pH 7.5; 300 mM NaCl; and 300 mM sucrose) (36). Lysis buffers were initially incorporated into and evaluated with a membrane-based commercial protocol (QiaAMP Viral RNA Mini Kit; Qiagen, Germantown, MD). Contrived DENV samples were either lysed with buffer AVL (as part of the kit) or an experimental buffer and then extracted with the remaining steps in the manufacturer’s protocol. Based on these experiments, the sucrose solution was chosen as the lysis buffer. NaCl, MgCl_2_, and KCl were evaluated as different chaotropic salts across a range of concentrations (50 to 400 mM) with varying sucrose concentrations (50 to 300 mM) and solution pH values (7.0, 7.5, and 8.0). Optimal lysis was obtained with a solution of 150 mM sucrose (Boston BioProducts, Ashland, MA); 50 mM Tris-HCl, pH 7.0; and 100 mM KCl (both from MilliporeSigma).

Poly-A carrier RNA (2.5 μg/sample; Qiagen, Germantown, MD) and proteinase K (5 μg/sample; New England Biolabs, Ipswich, MA) were evaluated as additional components to the lysis mixture. Further experiments used 25 μL/reaction of lysis mixture containing 17.5 μL of lysis buffer, 2.5 μL (2.5 μg) carrier RNA, and 5 μL (5.0 μg) proteinase K. Following preparation, lysis mixture was used immediately or stored at 4°C until use.

Arginine and glutamine amino acid buffers were initially prepared as described (38). Buffers contained 100 mM of amino acid (both from MilliporeSigma) and 400 mM KCl. pH was tested across a range of values from 1.5 to 9.1, with a final buffer pH of 1.5. Lysis and binding buffers were stored at room temperature for up to 6 months.

### Clinical samples and RNA stability.

DENV-positive clinical samples were collected as part of a study to detect and characterize arboviral infections in Asunción, Paraguay in collaboration with the Instituto de Investigaciones en Ciencas de la Salud, Universidad Nacional de Asunción (IICS-UNA) (4). This study was reviewed and approved by the IICS Scientific and Ethics Committee (P38/2020) and the Emory Institutional Review Board (study 00110736).

Samples were selected for the RNAES protocol clinical evaluation that had quantifiable DENV viral loads in the DENV multiplex rRT-PCR (42, 43) and had sufficient volume remaining for reextraction (100 μL). All samples had been shipped to Emory on dry ice, stored at −80°C, and thawed at 4°C immediately prior to extraction. Extractions were performed in duplicate with the RNAES protocol and once on an EMAG robotic extraction instrument (bioMérieux, Durham, NC). RNA was extracted from 25 μL of sample and eluted into 50 μL for both the packets and EMAG protocols. Eluates were tested immediately by rRT-PCR.

DENV-1 samples with sufficient remaining volume were individually reextracted to evaluate RNA stability on dried packet membranes on days 7 (*n* = 10) and 35 (*n* = 5) postextraction. Six extraction packets were prepared, with duplicate packets for each time point. On day 0, DENV RNA was completely extracted, eluted off two membranes, and run in the DENV multiplex rRT-PCR to establish a baseline. For the other time points, DENV RNA was extracted with the packets through the glycine wash step on day 0 and transferred to empty 1.5-mL tubes to air dry at ambient temperature for 1–3 hours. After drying, tubes were closed and stored in airtight plastic bags with desiccant packets. RNA was eluted from dried membranes with 50 μL TE buffer on days 7 and 35 following extractions. Eluates were run in the DENV multiplex rRT-PCR for comparison with day 0 results. A four-point standard curve was included on each run to calculate DENV-1 RNA concentration at each time point (42, 43).

### Reference viral RNAs and contrived samples.

Packet membranes were evaluated with viral RNA from DENV, CHIKV and Oropouche virus (OROV). DENV RNA for this portion of the study was a 135-base synthesized DENV-2 RNA oligonucleotide containing the DENV multiplex rRT-PCR target sequence (Ultramer RNA, Integrated DNA Technologies; Coralville, IA). For CHIKV and OROV, previously extracted (EMAG) genomic RNA was used. Contrived clinical samples were prepared by spiking negative human serum or plasma (MilliporeSigma) with DENV-positive serum of known concentrations. Aliquots of contrived specimens were prepared and stored at −80°C until use.

### rRT-PCR.

Eluates from optimization and analytical evaluation experiments were tested in a single-reaction multiplex rRT-PCR for ZIKV, CHIKV, and DENV or a single-plex rRT-PCR for OROV, both performed as previously described (44, 45). For the clinical evaluation, eluates were tested in the DENV multiplex rRT-PCR, which is a serotype-specific assay for DENV detection and quantitation (42, 43). All rRT-PCR reactions were performed in 20-μL reactions of the SuperScript III Platinum One-Step qRT-PCR kit (Thermo Fisher) containing 5 μL of eluate and run on a Rotor-Gene Q instrument (Qiagen). Positive and negative controls were included on each run, and rRT-PCRs were analyzed and interpreted as described previously (42–45).

### Statistics.

Basic statistical analyses were performed in Excel (Microsoft, Redmond, WA). *C_T_* values were compared by unpaired Student’s *t* test. The *t* tests, linear regression, and the Bland-Altman plot were performed using GraphPad Prism (version 9.2; GraphPad, San Diego, CA). Graphs were prepared with GraphPad and Excel.
