# Supplementary material for: Simple and Economical Extraction of Viral RNA and Storage at Ambient Temperature
Source: Microbiol Spectr. 2022 Jun 1;10(3):e00859-22. doi: 10.1128/spectrum.00859-22 (PMC9241768; doi:10.1128/spectrum.00859-22)
Supplement: Supplemental file 1 — Fig. S1 to S3, Tables S1 to S7. Download spectrum.00859-22-s0001.pdf, PDF file, 0.3 MB [file spectrum.00859-22-s0001.pdf]

Simple and economical extraction of viral RNA and storage at ambient temperature

Supplemental Material

Sarah Hernandez, Fátima Cardozo, David R. Myers, Alejandra Rojas, Jesse J. Waggoner

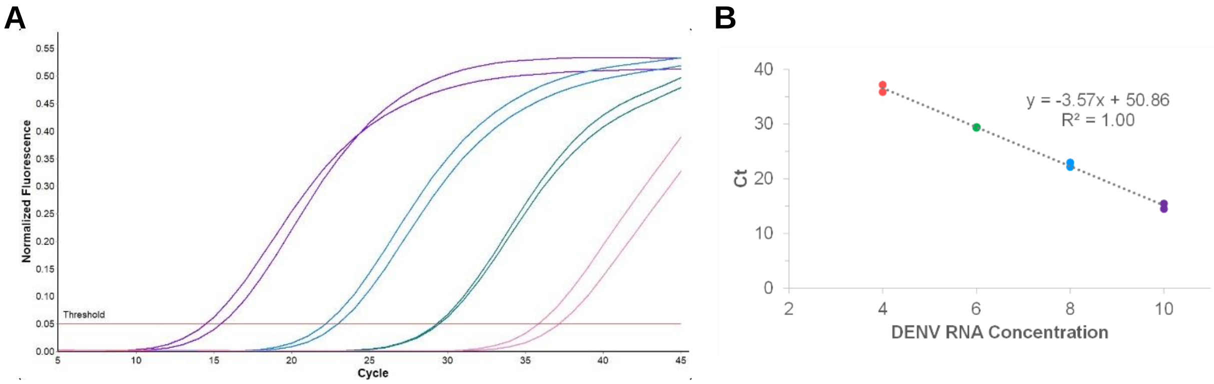

Figure S1. Concentration dependence of DENV RNA binding to RNAES packet membranes (Whatman 3 in this example). **A**) Amplification curves and **B**) linear regression of Ct values versus DENV RNA concentration of the solution added to sucrose lysis buffer. DENV RNA was tested at 10.0 (purple), 8.0 (blue), 6.0 (green), and 4.0 (pink) $\log_{10}$ copies/µL.

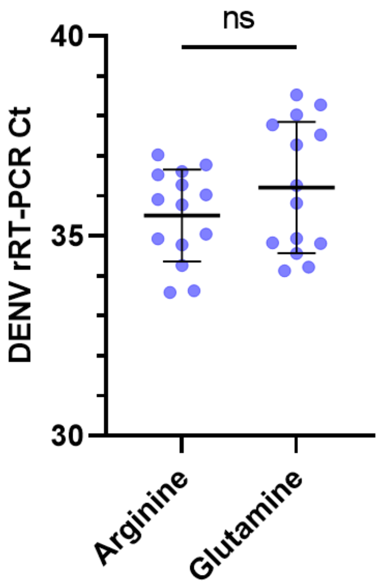

Figure S2. rRT-PCR cycle threshold (Ct) values for lysed DENV-positive serum samples treated with glutamine versus arginine binding buffer prior to addition to extraction packets. All evaluations were done with glass fiber GF/D membranes. Ct values were not significantly different for samples treated with arginine (mean, 35.51; standard deviation, 1.15) versus glutamine (36.21; 1.64; $p = 0.2$).

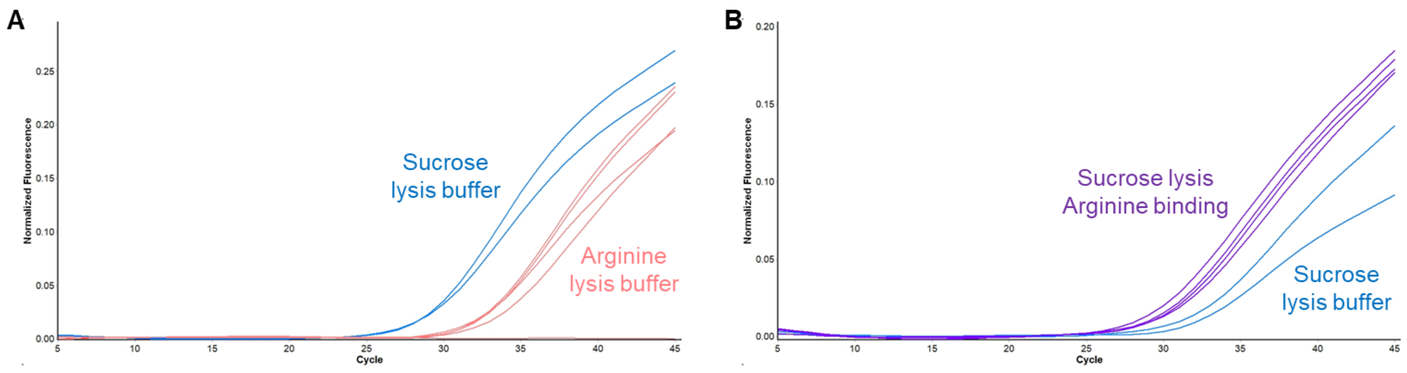

Figure S3. A) Use of arginine buffer for viral lysis results in reduced RNA yield from extraction packets. **B**) A combined protocol incorporating arginine buffer as a binding buffer after lysis in sucrose buffer results in increased RNA yield compared to an extraction protocol without a binding buffer. Different contrived samples were used on the two runs.

Table S1. Comparison of DENV Ct values following RNA extraction in experimental lysis buffers.

Buffer	Replicate	Ct
AVL [a]	1	26.36
	2	26.10
Deionized water	1	33.57
	2	34.07
Sucrose solution	1	28.15
	2	29.14
STET[b]	1	40.64
	2	39.25
SDS-NaCl	1	28.94
	2	28.53

[a] Guanidine thiocyanate-based lysis buffer in the QIAamp Viral RNA Mini Kit

[b] 8% Sucrose, 5% Triton™ X-100, 50mM Tris-HCl, and 50 mM EDTA

Table S2. Comparison of DENV Ct values following RNA extraction in lysis mixtures with and without carrier RNA.

**Replicate**	**Sucrose buffer only**	**Carrier RNA (2.5ug/sample)[a]**	**Carrier RNA (5.0ug/sample)[b]**
1	29.30	27.27	27.10
2	30.13	26.75	27.79
3	30.27	27.27	–
4	30.08	26.75	–
5	30.51	27.03	–
6	29.72	27.42	–
Average	30.00	27.08	27.45

[a] $p < 0.001$ for comparison with sucrose buffer only (unpaired t-test)

[b] "–" indicates not tested

Table S3. Comparison of DENV Ct values following RNA extraction in lysis mixtures with and without proteinase K.

Replicate	Sucrose buffer + carrier RNA	Proteinase K (5.0ug/sample)	Proteinase K (10.0ug/sample) [a]
1	32.09	28.34	28.38
2	29.50	27.12	27.24
3	31.14	27.31	27.38
4	33.00	26.23	26.92
5	32.46	26.48	–
6	32.71	26.35	–
Average	31.82	26.97	27.48

[a] $p < 0.001$ for comparison with sucrose buffer plus carrier RNA (unpaired t-test)

[b] "–" indicates not tested

Table S4. DENV rRT-PCR Ct values following sample incubation in lysis mixture for 10, 30, and 60 minutes at ambient temperature.

Lysis Incubation	***Ct Value***	***Average Ct***
10 min	31.14<br>34.30	32.72
30 min	34.55<br>35.65	35.10
60 min	33.85<br>33.78	33.82

Table S5. Comparison of DENV Ct values following extractions using sucrose lysis buffer containing 100mM $MgCl_2$ or KCl.

		**Sucrose Lysis Buffer**	
**Sample**	**Replicate**	**$MgCl_2$**	**KCl**
1	1	31.99	31.10
	2	30.90	31.33
2	1	34.73	34.66
	2	34.34	34.09

Table S6. Ct values following RNA extraction with economical packets and an EMAG commercial robotic system.

Sample code	Serotype	Packet Replicate 1	Packet Replicate 2	Average Packet Ct	EMAG Ct
16	D1	24.88	26.22	25.55	17.30
54	D1	28.65	28.60	28.63	23.65
60	D1	30.68	28.34	29.51	22.78
63	D1	27.02	26.92	26.97	21.27
65	D1	26.37	24.31	25.34	21.64
67	D1	26.41	27.48	26.95	21.92
69	D1	31.38	29.51	30.45	27.07
73	D1	25.47	25.29	25.38	21.88
80	D1	28.73	29.83	29.28	24.23
88	D1	23.32	23.31	23.32	18.03
89	D1	25.36	24.75	25.06	19.73
95	D1	35.42	34.31	34.87	29.58
99	D1	24.73	24.17	24.45	18.11
100	D1	27.71	29.13	28.42	23.46
109	D1	26.03	26.04	26.04	20.63
118	D1	25.83	28.00	26.92	22.53
121	D1	33.37	32.48	32.93	31.08
149	D1	25.99	26.49	26.24	21.94
167	D1	29.97	28.91	29.44	23.99
177	D1	30.55	32.17	31.36	28.26
2090	D4	28.66	27.70	28.18	24.81

2140	D4	33.11	32.18	32.65	24.14
2176	D4	25.89	26.90	26.40	24.20
2257	D4	35.46	34.92	35.19	32.98
2260	D4	32.61	32.58	32.60	38.44
2353	D4	33.24	32.91	33.08	35.30
2410	D4	33.09	31.86	32.48	31.17
2424	D4	31.86	32.49	32.18	30.09
2431	D4	35.12	33.90	34.51	33.15
2435	D4	33.22	32.99	33.11	38.36
2470	D4	29.39	29.56	29.48	26.52
2481	D4	39.01	39.06	39.04	34.95
2499	D4	30.80	30.54	30.67	28.30
8242	D4	N	36.39	36.39	38.29
8371	D4	36.11	39.53	37.82	37.07
8410	D4	38.29	39.44	38.87	40.50

Abbreviations: EMAG, commercial robotic extraction system (bioMérieux); N, negative

Table S7. Concentration of DENV RNA, presented as log_{10} copies/μL of eluate, following ambient temperature storage for 0, 7, and 35 days on dried membranes.

Sample Code	Day 0	Day 7	Day 35
54	3.51	3.27	-
63	4.37	4.03	-
67	4.43	4.57	-
88	5.50	5.10	-
99	5.28	5.22	-
65	4.30	3.92	4.03
89	4.22	4.19[a]	4.10
118	3.74	3.47	3.03
149	3.55	3.42	3.47
88/89 [b]	4.28	4.49	3.93

[a] Represents a single data point. One of two extractions failed at this concentration and time point.

[b] Contrived sample containing a mixture of 88 and 89.
